# Looking for trouble: a description of oculomotor search strategies during live CCTV operation

**DOI:** 10.3389/fnhum.2013.00615

**Published:** 2013-09-30

**Authors:** Matthew J. Stainer, Kenneth C. Scott-Brown, Benjamin W. Tatler

**Affiliations:** ^1^Active Vision Lab, School of Psychology, University of DundeeDundee, Angus, UK; ^2^Department of Optometry and Vision Science, University of MelbourneMelbourne, VIC, Australia; ^3^Department of Psychology, University of Abertay DundeeDundee, Angus, UK

**Keywords:** CCTV, surveillance, visual search, spatial selection, eye guidance, multiplex

## Abstract

Recent research has begun to address how CCTV operators in the modern control room attempt to search for crime (e.g., Howard et al., 2011). However, an often-neglected element of the CCTV task is that the operators have at their disposal a multiplexed wall of scenes, and a single spot-monitor on which they can select any of these feeds for inspection. Here we examined how 2 trained CCTV operators used these sources of information to search from crime during a morning, afternoon, and night-time shift. We found that they spent surprisingly little time viewing the multiplex wall, instead preferentially spending most of their time searching on the single-scene spot-monitor. Such search must require a sophisticated understanding of the surveilled environment, as the operators must make their selection of which screen to view based on their prediction of where crime is likely to occur. This seems to be reflected in the difference in the screens that they selected to view at different times of the day. For example, night-clubs received close monitoring at night, but were seldom viewed in mid-morning. Such narrowing of search based on a contextual understanding of an environment is not a new idea (e.g., Torralba et al., 2006), and appears to contribute to operator's selection strategy. This research prompts new questions regarding the nature of representation that operators have of their environment, and how they might develop expectation-based search strategies to countermand the demands of the large influx of visual information. Future research should ensure not to neglect examination of operator behavior “in the wild” (Hutchins, 1995a), as such insights are difficult to gain from laboratory based paradigms alone.

## Introduction

The task of the CCTV operator is to find and, if possible, prevent crime in public spaces. Research has shown that when asked to predict whether the events presented in a single video will turn violent, naive observers perform as well as trained CCTV operators (Troscianko et al., 2004; Grant and Williams, 2011). In control rooms, however, the CCTV operator is tasked with searching for such undefined targets across not one, but a vast number of screens displaying dynamic information from locations across a wide geographical area. For example, in a survey of 11 local authority and private security CCTV control rooms, operators were faced with a range of 27–520 cameras per operator, with up to 175 feeds displayed simultaneously across a bank of monitors (Gill and Spriggs, 2005; Gill et al., 2005). As such, the visually rich layout of the modern CCTV control room seems unnatural, complex, and ill-suited to the perceptual and cognitive constraints of the human operator (Scott-Brown and Cronin, 2008). It is well characterized that when searching for a target, the number of distractors that are present can dramatically influence search time (see review by Wolfe, 1998), including when a target's identity is not known (Rensink, 2000). Thus, performance skill in CCTV operation may be better characterized by their ability to find a “target” scene (i.e., containing information for the task) amongst a large number of “distractor” scenes (e.g., see Howard et al., 2009).

### Multiple scene surveillance

Tickner and Poulton (1973) demonstrated the behavioral costs when faced with increasing numbers of scenes in a surveillance-based task. These authors showed that when monitoring simultaneous feeds from cameras in a prison, the accuracy with which participants detected suspicious events was lower when the number of simultaneously-viewed camera feeds was high; with 83% for 4 monitors, 84% for 9 monitors, and 64% for 16 monitors. Wallace et al. (1997) examined observers' target detection across multiple scenes and found decreases in performance when increasing the number of town center scenes in the display. Correspondingly, this difficulty is reflected in CCTV operators confidence of multi-scene detection. When interviewed, 82% of CCTV operators interviewed only reported confidence with monitoring up to sixteen screens, with 50% reporting that they felt comfortable monitoring up to four screens simultaneously (Wallace and Diffley, 1998). This is considerably less than the number of screens that can be displayed in the modern control room. In another study, Howard et al. (2011) presented participants with a series of quadraplex displayed CCTV clips and recorded their perceived suspiciousness of the video by means of a joystick. Participants moved the joystick forward to indicate their belief that an event was likely to happen. Viewers eye-gaze in these conditions, where multiple different video streams compete for attention, was allocated according to the relative suspiciousness of each video clip.

**Figure 1:**
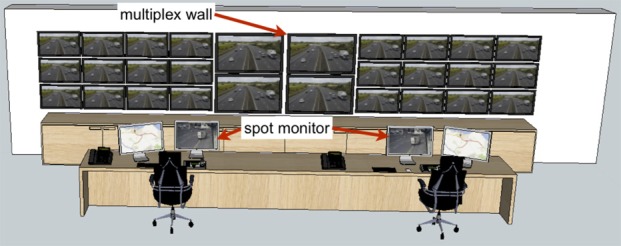
**Prototypical layout of modern provincial CCTV control room layout similar to that used by the Tayside Police**. 3D model adapted from Google SketchUp program by artist “STUFF & STUFF.” Each operator has their own controllable spot monitor, an additional monitor, a computer keyboard a camera control keypad and a telephone headset to wear. Metropolitan area control rooms may feature more operators and a larger array of screens on the wall.

The overriding message from what is known about visual information load and visual search performance (e.g., Wolfe, 1998), and the performance in multiple-scene detection tasks (Tickner and Poulton, 1973; Howard et al., 2011) is that efficient search for crime among a large number of scenes is likely to be poor. However, while simultaneous display of a large number of camera feeds in multiplexes is an integral feature of CCTV control rooms, operators also have at their disposal individual spot monitors that can be used to selectively view the information from a single camera at a time (Figure 1). The selection of content in this way is an often-neglected element in studies of the CCTV task and it is important to characterize the relative use of multiplex and spot monitor for real surveillance situations.

Not only is it important to understand the manner in which the multiplex and spot monitor are used in surveillance, but it is also important to consider the different cognitive demands associated with the use of each of these display formats. In the multiplex, all visual content is displayed at one time. However, skilled and strategic use of the spot monitor relies on an understanding of the camera array and geographical area under surveillance, (Hillstrom et al., 2008). For example, tracking a suspect across an extended area of space requires selection of geographically adjacent cameras, even though they may neither be spatially adjacent in the multiplex nor visually continuous in content. Thus, selection on the spot monitor is not simply based on visual guidance, but rather by the representation of the environment or a mixture of the two. The multiplex and the spot monitor therefore present rather different challenges and opportunities for the operator and potentially rely on rather different underlying knowledge. Moreover, these two display types may be differentially suited to particular aspects of the surveillance task: the multiplex might be well suited for detecting unexpected or suspicious events as these might occur in any of a number of different locations in the environment at any time. On the other hand, detailed information of unfolding events at a particular location might be better accessed via the spot monitor, where potential distraction from other camera feeds can be avoided.

One relatively unexplored aspect of the surveillance task is the extent to which the task demands vary over a 24-h period and how this impacts on operator behavior. For example, flash-point outbreaks of violence are a more prominent feature of the task at night than during the day in many urban settings (Felson and Poulsen, 2003). Not only do the likely types of events differ over the 24-h period, but also the likely locations at which these events occur changes: night-clubs are a likely venue for fights at night, but not during the day. During a visual search task, when people are told the area of a scene that contains the target, performance is related to the size of that area, rather than the whole scene (Zelinsky and Schmidt, 2009). In the control room, and idea of where to look for different targets would likely serve to reduce the load of the observer. Similarly contextual understanding of scenes has been shown to influence where people search for items (Torralba et al., 2006). Given the intimate link between vision and task demands in real world activity (see Land and Tatler, 2009) it seems likely that visual strategies of CCTV operators will vary depending upon the time of day or night during which they are working.

The cognitive ethology (Kingstone et al., 2008; or ethnography e.g., Hutchins, 1995a; Hollan et al., 2000) approach to understanding how a system functions can provide otherwise hidden insights into how tasks are completed, such as how drivers navigate corners (Land and Lee, 1994). The purpose of this paper is to offer a first step toward understanding the nature of the surveillance task as it exists in a real CCTV control room. While in doing so we sacrifice some of the control which laboratory paradigms afford, such studies are essential to ensure that the questions we can ask in the lab are valid to the task (see also Hutchins, 1995a and Weibel et al., 2012 for a recent example including eye-tracking).

The first question we examined was to look at what extent the operators use the multiplex or the spot monitor. Research has addressed both single scene (e.g., Troscianko et al., 2004) and multiplex surveillance (e.g., Tickner and Poulton, 1973) viewing conditions, but a systematic analysis of their use in day-to-day Control Room operation has yet to be conducted. The second question that this paper addresses is to what extent is selection based on the monitoring task, and, by extension, to what extent is selection based on the viewing preference of the individual operator? If the task dictates spatial selection, then we would expect there to be larger differences in selection of content between shifts of operation. However, if selection is more related to the preferences of the individual operator, we would expect selection to be more different between the operators, and similar across different sessions.

## Materials and methods

### Participants

The observers were two trained CCTV Control Room operators from Tayside Police (now “Police Scotland”) Control Room. Operator 1 had been working as an operator for approximately 10 years, whereas Operator 2 had been in the position for approximately 2 years (and was trained by Operator 1).

### Tayside control room

**Figure 2:**
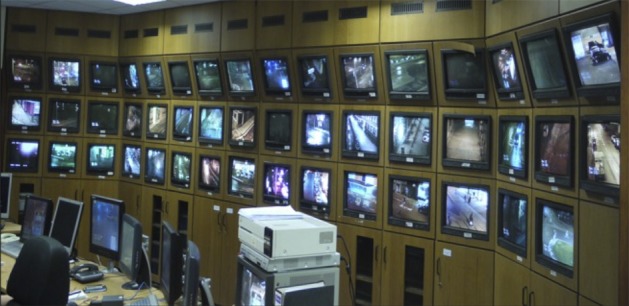
**Tayside Police control room**.

Tayside Police Control Room receives live feeds from around 100 CCTV cameras in the Dundee City area at any one time. These camera feeds are displayed on a multiplexed bank of 47 CRT monitors (Figure 2). Several of the monitors are used to simultaneously display four camera feeds in split-screen (usually low-activity scenes such as car parks), and some automatically scroll through up to five different cameras, showing each one at a time for a period of several seconds. Many of the cameras are also on a set “walk” pattern, whereby they automatically pan across the area in a pre-set manner. Both operators that we recorded reported being able to comfortably see detail on the multiplex from their viewing position.

Operators in Tayside Control room work in teams of two (although they may occasionally be joined by a third person who will review footage on a separate station). This research was authorized by the Force Executive of Tayside Police.

### Eye movement recording

Eye movements were recorded using a lightweight Positive Science LLC mobile eye tracking system built by Jason Babcock (Babcock and Pelz, 2004). The system samples eye position at a 30 Hz and creates a video overlay of the scene viewed from a first person perspective with a gaze-cursor cross. Two cameras were mounted on a spectacle frame, simultaneously recording the scene and the observer's eye. The key benefit of this system is its unobtrusive qualities. Thanks to its small visual footprint and low-weight construction, operators can enjoy full freedom of movement in their normal seated position. As viewing behavior may be influenced by the process of wearing an eye-tracker (e.g., an “eye-tracker awareness”; Risko and Kingstone, 2011), operators were given no instruction other than to carry out their task as usual to attempt to minimize experimenter effects.

The video from the cameras was captured live into the Yarbus software package (version 2.2.2) on a MacBook Pro (4 GB Memory, 2.4 GHz Intel Core 2 Duo), where eye position was estimated based on detection of the pupil (with accuracy within a degree of visual angle). Observers calibrated live using a 9-point grid made up of the corners of monitors on the data wall, and the four corners around their spot monitor.

**Figure 3:**
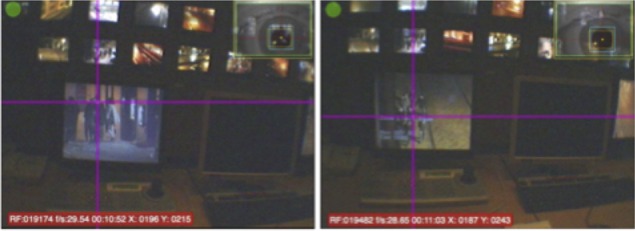
**Examples of eye gaze videos with gaze position crosshair overlaid**.

Data were exported as videos from the scene camera overlaid with eye position (Figure 3). The videos were then hand-coded to extract where the operators were looking throughout each session in terms of the type of display (multiplex and spot-monitor), and the camera feed that was shown on that display. Data during blinks were excluded from analysis.

### Procedure

Recordings were made during live system operation from each observer at each of their three shifts of work (afternoon, morning, and late night). Each recording session for each operator was 15 min in duration. Care was taken to ensure fitting and removal of the equipment from the operator was performed at convenient times within the surveillance task so as not to interrupt actions *en train*. Operators were told that they could remove the glasses at any time if they felt it was hindering their work (although neither chose to at any point).

### Analysis

Our approach to examining the question of how operators search for crime is not a traditional experimental design, but rather an observational approach. There are potential issues with over-generalizing the data from such observations (particularly given the low number of operators). However, our aim is to describe behavior as it occurs. Thus, we applied traditional quantitative techniques of analysis to attempt to quantify this behavior, and describe the operators' use of the multiplex and the spot monitor in their search for crime. As such, some data presented are simply numerical (such as the number of cameras that an operator viewed on a particular session).

When examining differences between operators based on continuous variables, we used linear mixed-effect modeling (for example, to examine the difference in scanning time per scene between operators). Linear-mixed effects models have become increasingly used to examine non-normally distributed data (e.g., see Druker and Anderson, 2010). They allow for modeling of fixed factors, and random factors, with all data included (rather than condensing the data to a single mean). Thus, it considers the variance within a random factor (such as participant), as well as the variance between fixed factors. However, here we consider operator as a fixed factor. Conventionally the fixed factor in an analysis must be repeatable (Baayen, 2007, p263). However, we include operator here as a fixed factor, as we do not intend to generalize our data beyond differences between our operators (and simply to try to quantify if they *were* different).

Here, we analyzed the data using the *lme4* (Bates, 2005) and *languageR* (Baayen, 2007) packages in *R* (R Development Core Team, 2009). We follow the reporting style of Druker and Anderson (2010), who used similar modeling, to report the mean difference between conditions with highest 95% posterior density intervals from Markov Chain Monte Carlo mean estimates, with approximated *p* values generated with the *pval.fnc* function (Baayen, 2007; Baayen et al., 2008).

When looking at categorical differences between operators, we employed Kullback-Leibler divergence analysis (for example, to analyze whether there was a difference in the cameras selected between operators, and between sessions). Kullback-Leibler divergence is an information theoretic measure that allows us to quantify the difference between two probability distributions in terms of the number of bits of code that is required to describe one distribution based on another. We present these probability distributions in graph form, with camera number being a categorical factor, plotted against probability of fixation. Thus, the Kullback-Leibler divergence score can be used a measure of the difference between two categorical distributions (for similar use see Tatler et al., 2005). This allows us to quantify whether differences in selection are greater between shifts of operation, or between operators, with higher scores representing larger differences.

## Results

Across all sessions, we found that operators spent the majority of their time selecting content on their individual spot monitor (>90% across both observers in all sessions; Table 1). Operator 1 did not use the multiplex at all in the morning, or evening sessions, with the highest proportion of time spent on the multiplex being the afternoon session for both operators.

**Table 1:** **The proportion of time spent by each operator viewing their spot monitor, and the multiplex**.

		**Afternoon (%)**	**Morning (%)**	**Evening (%)**
Operator 1	Spot monitor	98.5	100	100
	Multiplex	1.5		
Operator 2	Spot monitor	91.51	97.57	98.47
	Multiplex	8.31	4.43	1.53

### Spot monitor scanning

**Figure 4:**
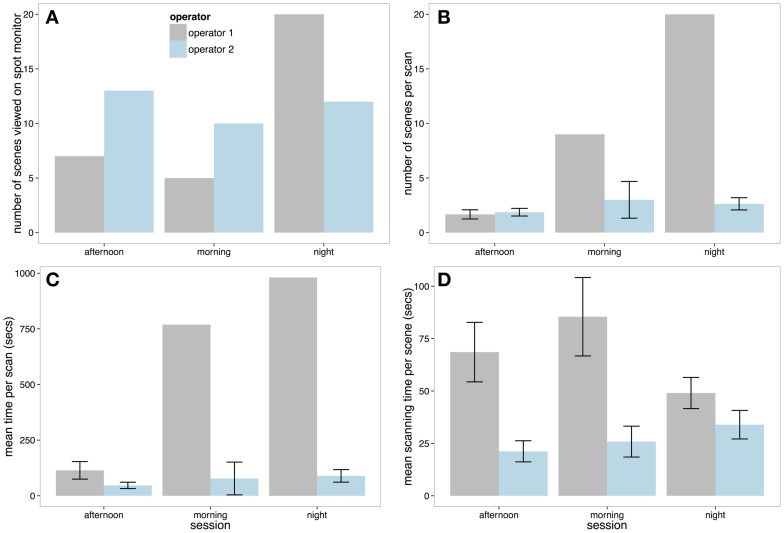
**(A)** Total number of scenes viewed on the spot monitor on each session by each operator. **(B)** The number of scenes selected by each operator per scan. This figure shows that Operator 1 selected more screens than Operator 2 (and this was unbroken in the morning and night session with no looks at the multiplex—hence lack of ±SE). **(C)** Mean length of each spot monitor scanning session. **(D)** Mean scanning time per scene on the spot monitor (with ±SE).

We looked at four principle measures of spot monitor use that are summarized in Figure 4. First, Figure 4A reveals that in the afternoon and morning sessions, Operator 1 viewed around half the total number of scenes compared to Operator 2. However, in the night session Operator 1 viewed more scenes in total than Operator 2 (although this total was less than the number of scenes viewed by Operator 2 other two sessions).

Per scan (a viewing session on the spot monitor that was uninterrupted by looks at the multiplex), Operator 2 was relatively consistent, viewing around 2–3 scenes between looks to the multiplex in all three shifts (Figure 4B). However, as Operator 1 did not view the multiplex at all in the morning and evening recording sessions, they viewed more scenes per scan than Operator 2. In the only session that Operator 1 did use the multiplex (the afternoon session), the number of scenes per scan was similar to Operator 2 (2–3 scenes). Correspondingly, Figure 4C shows that the Operator 1 had longer periods of spot monitor scanning than Operator 2 in all sessions.

Finally, we looked at how long Operators would view each scene before selecting to view content from a different camera. To examine scanning time per scene, a linear mixed effect model was carried out with operator included as a fixed factor, and session included as a random factor. Figure 4D demonstrates that Operator 2 inspected each scene for significantly less time than Operator 1 (Markov-Chain Monte Carlo (MCMC) mean difference = −35.19 s, 95% *CI* = −49.86 to −21.55 s, *p* < 0.0001).

### Spot monitor selection

**Figure 5:**
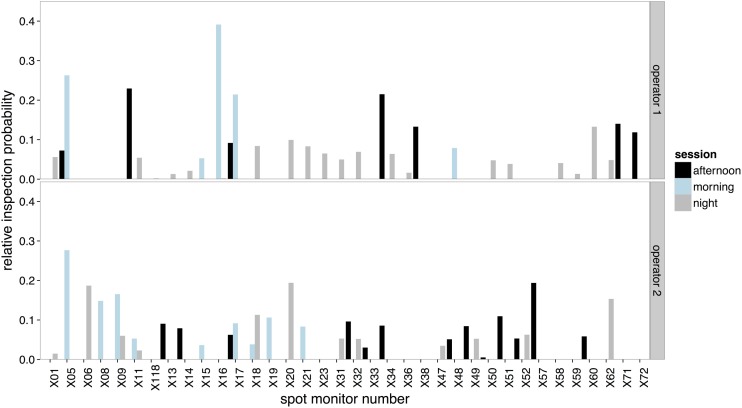
**The relative inspection probabilities for each scene selected on the spot monitor by Operator 1 (upper panel) and Operator 2 (lower panel)**.

**Figure 6:**
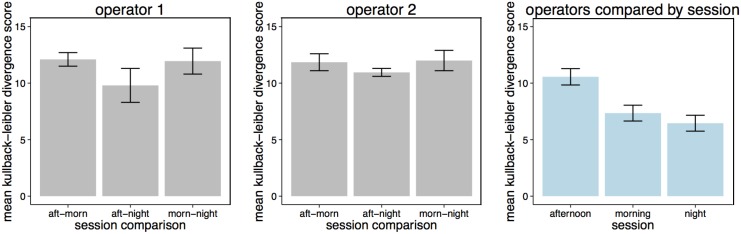
**Kullback-Leibler divergence score in screens selected for viewing on the spot monitor by session for Operator 1 (panel 1), Operator 2 (panel 2) and across all sessions (panel 3)**. ±SE are included, and represent that Kullback-Leibler divergence analysis gives two scores for each comparison (the probability of distribution A/B, and the probability of distribution B/A).

The amount of time that operators spent on each selected scene viewed on the spot monitor across the three recording sessions is illustrated in Figure 5. Operators' selection of content was most similar between the afternoon and night sessions (Figure 6 center bar of panels 1 and 2). Operators showed the greatest difference in the scenes that they chose to view on the spot monitor in the morning compared to the night shift (right bar of panels 1 and 2). The scenes that were selected at night were most similar between operators (right bar of panel 3), and least similar in the afternoon.

### Multiplex scanning

**Figure 7:**
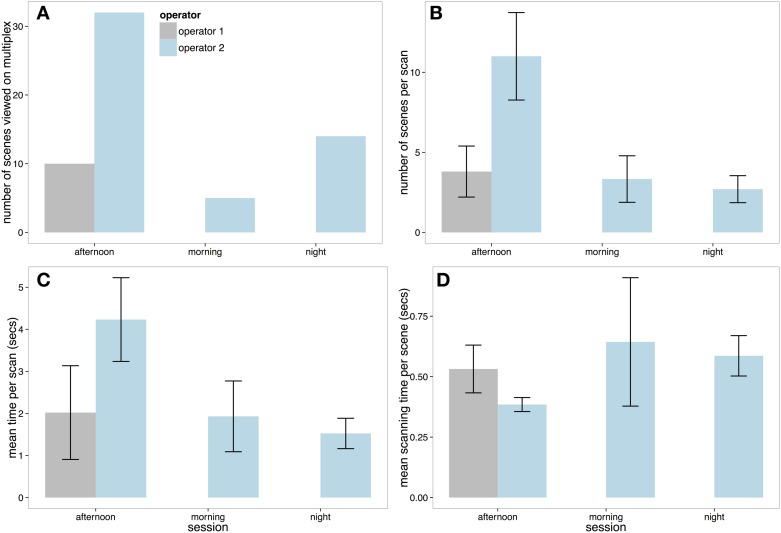
**(A)** Total number of scenes viewed on the multiplex on each session by each operator. **(B)** The number of scenes selected by each operator per scan. **(C)** Mean length of each multiplex scanning session and **(D)** Mean scanning time per scene on the multiplex (with ±SE).

As discussed previously, Operator 1 used their spot monitor for the entire morning and night session. Figure 7A reveals that Operator 2 viewed just over 3 times as many scenes in the afternoon session compared to Operator 1. Operator 2 also viewed more scenes per scan (Figure 7B), and had longer periods of multiplex scanning (Figure 7C). However, Figure 7D reveals that when Operator 1 did look at scenes on the multiplex, the operator spent more time on average viewing each scene before moving to another.

### Multiplex selection

**Figure 8:**
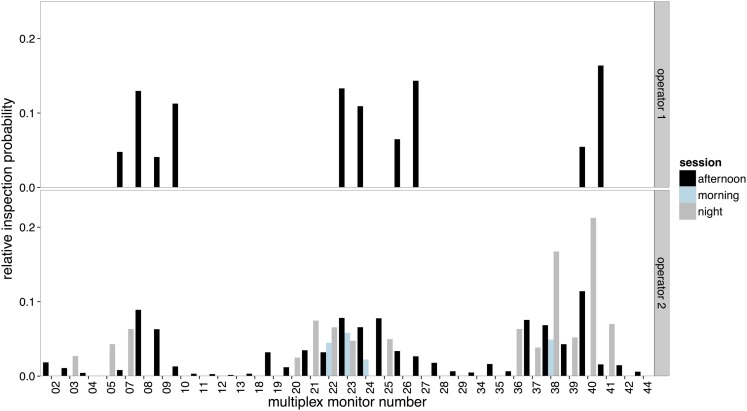
**The relative inspection probabilities for each scene selected on the multiplex by Operator 1 (upper panel) and Operator 2 (lower panel)**.

**Figure 9:**
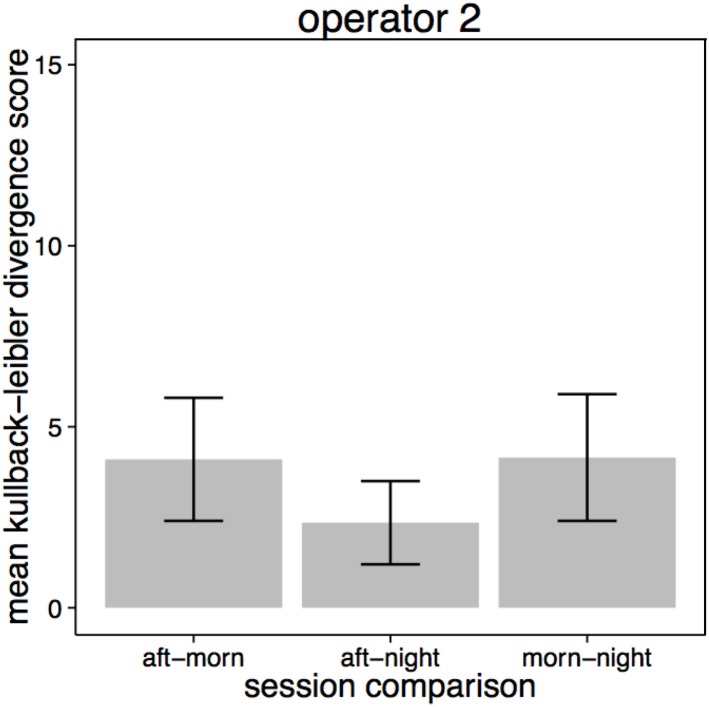
**Kullback-Leibler divergence score in screens selected for viewing on the multiplex by session for Operator 2**.

The distributions of time spent viewing scenes on the multiplex can be seen in Figure 8. As Operator 1 did not use the multiplex on either the morning or afternoon session, only selection by Operator 2 was examined using Kullback-Leibler divergence. Figure 9 shows that there was much less variance in selection on the multiplex between sessions compared to the content viewed on the spot monitor (which yielded higher Kullback-Leibler scores). However, when compared across sessions, selection followed a similar pattern as on the spot monitor. Selection of content was most similar between the afternoon and night sessions.

## Discussion

In what we believe to be the first study of visual strategies for expert CCTV surveillance in a public space control room under normal working conditions, we report the results of a mobile eye-tracking study of CCTV operator performance during day and night shift team-based surveillance. Spot monitor scanning and selection was compared with multiplex scanning and selection data along with a comparison of inter-operator differences in screen inspections.

For the operators we studied, spot monitor observation took up more than 90% of inspection time in the control room during the periods of observation (afternoon, morning, and evening). The data demonstrate that during our recording spatial selection in the control room differed dramatically both between operators, and between different shifts of operation. For example, Operator 1 spent more time viewing content on the spot monitor than Operator 2, and spent longer on each scene before transitioning. These differences between operators may reflect different idiosyncratic styles for surveillance or the differing experience of the two operators. However, the operators work as a team and these differences may reflect the different roles that each operator took in their collaborative effort. For example, Operator 1 might take the role of monitoring the night clubs, while Operator 2 monitors at the suburbs. Such distribution of cognition has been previously demonstrated, for example, between pilots in the cockpit of an airplane (Hutchins, 1995b). While the question of how operators work together to efficiently detect crime was not the aim of this study, this would likely be an informative and interesting direction for future research.

Despite the data showing that during three 15-min recording sessions the operators spent little time viewing content on the multiplex, when operators did use the multiplex, they were more similar to each other in what they chose to view compared to their selections for inspection on the spot monitor. Short scans of the multiplex lasting approximately 1–4 s punctuate the longer spot monitor views, and inspection times for individual scenes are extremely short when viewed on the multiplex. Thus, it appears that anything worth further inspection is probably brought to the spot monitor, and multiplex viewing may be used primarily to help identify content that should receive more detailed scrutiny. Content selection in the multiplex appears most similar in afternoon and night conditions.

These findings indicate that approaches to understanding surveillance that are based solely on multiplex detection (Tickner and Poulton, 1973) or single screen detection (Troscianko et al., 2004) may provide insights into aspects of the task. However, given the dynamic interplay between multiplex viewing and selecting single camera feeds for further inspection, these two modes of viewing need to be considered together. Moreover, single screen viewing is a very active process in which content from different cameras is actively selected, with new camera feeds being selected on average every 26.94 (Operator 2) to 62.44 (Operator 1) s while using the spot monitor. Selection of content during spot monitor use necessarily reflects considerable use of the internal representation of the surveilled environment, including an understanding of the camera locations in external space.

### Strategies in searching for crime

When searching for crime, we found that the CCTV operators spent very little time searching the multiplex. In accordance to this finding, operators of multiplex systems reported low confidence in their ability to monitor several scenes (Wallace and Diffley, 1998). This would be entirely consistent with what is understood about search of complex displays (e.g., see Wolfe, 1998). Increasing the amount of visual information in a display increases search time (with visual information measured in several methods; Rosenholtz et al., 2007; Henderson et al., 2009; Beck et al., 2010; Bi et al., 2010; Wolfe et al., 2011; Asher et al., 2013). Given the likelihood of a bottleneck of attention at some-point in the visual system (for example, see limits on the number of objects we can simultaneously track; Alvarez and Franconeri, 2007), the multiplex might present a daunting task to the visual system. Performance drops have been shown at four screens (Tickner and Poulton, 1973, or Rousselet et al., 2004), which was less than 1/10th of the screens in the multiplex of the control room examined here.

One way that operators might effectively be able to increase confidence is to use the spot monitor (i.e., reduce the task to a single scene load). If operators conduct the majority of work on their single spot monitor, it is important that they select the appropriate scenes to view. While previous research has found no effect of training on single scene detection tasks (such as Troscianko et al., 2004), it may be that expertise in the control room serves to guide operators' search for crime within the large number of scenes that they could potentially select and view. Accordingly, Howard et al. (2010) demonstrated that the difference between experts and novices watching a five-a-side football match is that experts look at the most informative locations earlier than novices. In the surveillance context, we found that operators appear to select content differently at different times of day and this seems likely to be based on both their knowledge of the environment and their experience of where events are likely to occur at different times of the day.

It is important to consider how operators are able to select a subset of appropriate content from the large array of camera feeds available. It is possible that this is based on reactive selection to events unfolding in each camera feed. However, the proactive nature of surveillance and the often subtle events that are selected for detailed monitoring suggests that the selection processes are likely to be strategic, based on prior knowledge and expectation. One plausible possibility is that operators have an understanding of the likely locations at which events will occur at different times and that they use this to constrain much of their surveillance effort to the cameras that depict these locations. In this way, suspicious events will be monitored primarily within expected locations in the surveilled environment. This suggestion is similar to the contextual selection that has been demonstrated in scene viewing paradigms, where observers appear to combine expectations of where things are likely to be in the world with low level feature information (Torralba et al., 2006; Ehinger et al., 2009). In such paradigms, it has been shown that observers primarily search regions in which targets are expected to occur, with search time being related to the area the observer has to search, rather than the whole display (Zelinsky and Schmidt, 2009). Some cameras facing night-clubs (e.g., feed X20 and X62) were not viewed at all in the morning and afternoon session, but made up a large proportion of the night-time surveillance. How operators develop their criteria for selecting appropriate content is a question that further research should seek to address.

We propose four potential ways that expectation might develop: First, expectation may simply be based on general associations of social factors (e.g., areas associated with drug use are more likely to be high violence areas, Lum, 2011). Second, expectation might be built up via reinforcement, as operators successfully experience or detect events in certain scenes (similar to the development of spatial bias in visual search, e.g., Carpenter and Williams, 1995). Third, it may be based on how the amount of activity (and hence content and motion within the camera feeds, e.g., see Howard and Holcombe, 2010) changes throughout the day. There are simply more people around night-clubs at night than anywhere else. Fourth, strategic selections may arise as a result of explicit instruction about where to look and when during operator training (e.g., Wallace and Diffley, 1998, Appendix A). We might speculate that the fourth possibility does not account for aspects of our findings because the two operators differed in the scenes they viewed, however, as previously suggested this difference might be an active choice for efficient collaboration of efforts across the control room.

### Conclusions

Research has shown that when observers attempt to detect criminal activity in one scene, untrained observers are as good as trained CCTV operators (Troscianko et al., 2004; Grant and Williams, 2011). However, this situation only captures one part of the CCTV operator's task. First, operators have to correctly select the scene to view from a large number of possibilities. As such, the task of CCTV operation is not simply a case of looking at the right place at the right time, but rather of looking at the right place at the right time *in the right scene*. To complete this task, we found that two trained CCTV operators spent more time searching for crime using a single scene spot monitor, rather than the multiplex data wall, despite the latter giving the operator more information at one time. This may, in part, reflect the difficulty of search across large amounts of visual information (e.g., Wolfe, 1998 among others). However, to be able to search with the spot monitor, operators must select screens based on their representation of the surveilled world. Moreover, this understanding of the environment seems to incorporate the monitoring demands associated with different shifts of operation, with operators selecting different screens at day compared to night, for example. This may reflect the locations of high event likelihood being different at night, compared to during the morning, which would be consistent with using contextual understanding to guide visual search to areas likely to contain a target (such as Torralba et al., 2006).

Using cognitive ethology, we can gain a more comprehensive, ecologically valid idea of how cognition functions “in the wild.” We echo the sentiments of Kingstone et al. (2008) that observation of naturally occurring behavior can provide an essential complement to laboratory-based studies in generating valid hypotheses and questions, as neither alone can provide a complete picture of complex cognitive tasks such as CCTV operation.

## Ethics statement

This research was carried out in accordance with, and approval of the University of Dundee Ethics Committee.

### Conflict of interest statement

The authors declare that the research was conducted in the absence of any commercial or financial relationships that could be construed as a potential conflict of interest.
